# Anaesthetic management of an adult patient with DOOR syndrome: a case report

**DOI:** 10.1186/1757-1626-2-7593

**Published:** 2009-05-18

**Authors:** Pavel Michalek, William Donaldson, Alexander Abraham

**Affiliations:** 1 Department of Anaesthetics, Antrim Area Hospital45 Bush Road, Antrim, BT41 4RDUnited Kingdom/Northern Ireland; 2 Department of Anaesthesia, Ulster HospitalUpper Newtownards Road, Dundonald, Belfast, BT16 1RHUnited Kingdom/Northern Ireland

## Abstract

We report the anaesthetic management of a 48-year-old male patient with Deafness, Onycho-Osteodystrophy and mental Retardation syndrome, epilepsy and cerebral palsy who had two dental procedures under anaesthetic care. For the first short examination sedoanalgesia was employed and the second, longer, procedure was performed under general anaesthesia. His airway management was moderately difficult and the postoperative period was complicated by partial seizures involving the upper extremity and a short period of decreased oxygen saturation. The potential anaesthetic implications of Deafness, Onycho-Osteodystrophy and mental Retardation syndrome are highlighted.

## Introduction

D.O.O.R. syndrome (acronym: congenital ***d***eafness, ***o***nychodystrophy, ***o***steodystrophy and mental ***r***etardation) was first described in 1961 [1] and eventually defined by Cantwell in 1975 [2]. The syndrome is a rare genetic disorder characterized by total or partial congenital bilateral sensorineural hearing loss resulting from anatomical malformation of the inner ear or auditory nerve, malformations of distal parts of fingers or toes [3], multiple malformations of the nails and mental retardation of varying degrees. Other features of the syndrome include craniofacial abnormities, neurological manifestations including epileptic seizures, optic atrophy and peripheral polyneuropathy [4,5]. The syndrome is most likely inherited in an autosomal recessive fashion and the majority of subjects have increased levels of 2-oxoglutarate in the plasma and urine [6]. Less than 50 cases have been reported in the medical literature and no article has been related to the anaesthetic management of patients with this syndrome.

## Case presentation

**Figure 1.**
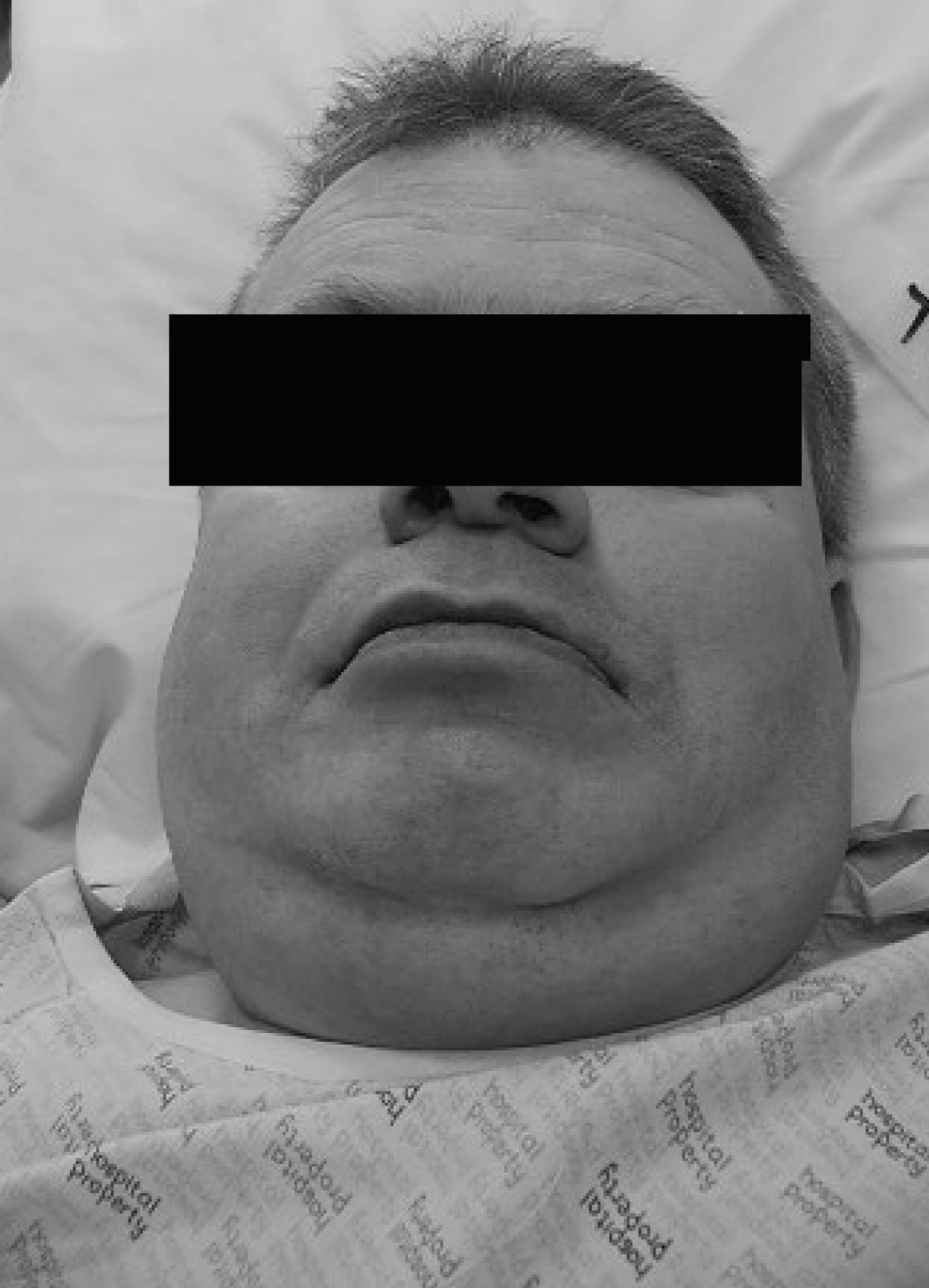
Facial features of the patient with DOOR syndrome.

**Figure 2.**
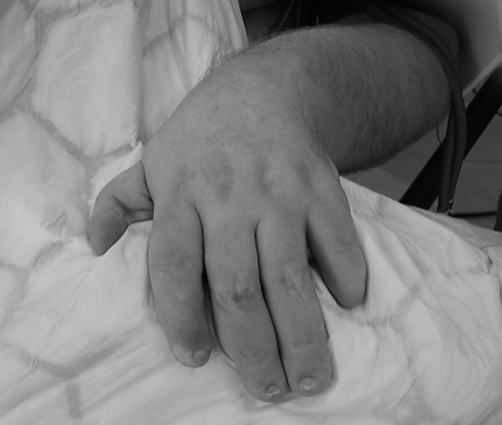
Onycho-osteodystrophy in DOOR syndrome.

A 48-year-old Caucasian man, weighing 108 kg (BMI 38) (Figure 1) was scheduled for dental treatment under general anaesthesia. He had been diagnosed with DOOR syndrome as an infant and had typical features, for example: malformations of distal phalanges of the fingers and nails (Figure 2). Other manifestations during childhood included epilepsy with frequent grand-mal seizures, deafness and significantly delayed psychomotor development. Aside from features associated with DOOR syndrome and obesity, the patient also had left sided cerebral palsy, recurrent chest infections, asthma, gastroesophageal reflux and hypertension. It was difficult to elucidate any history of obstructive sleep apnoea due to his learning difficulties. There was no cardiac defect or urogenital abnormities. His medication included carbamazepine, omeprazole, salbutamol and budesonide. He had no allergies. At anaesthetic preassessment the only findings noted were a thyromental distance of 7.5 cm and good neck mobility. The neck circumference was measured as 40 cm. Further airway assessment, such as the Mallampati score, was impossible. Basic observations were performed, including a blood pressure reading of 150/90 mmHg and a heart rate of 72 bpm. Oxygen saturation on room air was 95%. He was extremely uncooperative, rendering an awake dental examination unfeasible. A plan was therefore made to use short sedation for dental assessment including X-rays and then to administer general anaesthesia for definitive treatment. The patient was premedicated with 4 mg of buccal midazolam and sedated with titrated intravenous midazolam to a total dose of 5 mg and fentanyl 100 mcg. Depth of sedation was assessed using entropy monitoring (GE Healthcare, Chalfont St. Giles, UK). The baseline values of entropy were inside the normal range - SE 87, RE 98. During intravenous sedation the levels dropped to 60 (SE) and 75 (RE) which corresponded with values seen in patients without learning difficulties. The procedure lasted 25 minutes and was uneventful. Based on X-ray examination, the patient was scheduled for multiple extractions and fillings under general anaesthesia. The patient was fasted for 6 hrs, and had his usual dose of antiepileptic and inhalers. Premedication with 4 mg of buccal midazolam was given, after which a 20 G i.v. cannula was placed and ranitidine 50 mg and metoclopramide 10 mg administered intravenously. We routinely use these drugs in learning disability patients as the fasting interval may not be adhered to. Induction was with propofol (2.5 mg.kg^−1^) and fentanyl (1 mcg.kg^−1^). After a trial of bag mask ventilation, atracurium 0.5 mg.kg^−1^ was administered. Due to his specific facial features and short neck a McCoy blade was used in the first instance. The laryngeal view was a Cormack and Lehane grade II and the trachea was intubated at the first attempt with a size 8.0 cuffed RAE tube and a cotton throat pack was inserted. The difficult airway trolley was available and our plan ‘B’ involved the insertion of a laryngeal mask airway. Maintenance of anaesthesia was with sevoflurane in an air/oxygen mixture. Further doses of fentanyl were administered for analgesia to a total dose of 200 mcg. The procedure lasted 100 minutes and included two complicated surgical extractions and four fillings. The dentist infiltrated local anaesthetic. The patient was extubated fully awake without complications. In the recovery ward, twenty minutes after extubation, he developed mild partial seizures affecting his right forearm and hand. At the time of these seizures, the patient was fully awake, with SpO_2_ 97% on oxygen mask, and was haemodynamically stable. The seizure activity lasted 10 minutes and abated spontaneously. Other postoperative complications included a short period of decreased saturations and moderate nausea. The patient was discharged home that same evening, 4 hours post-procedure. Oral paracetamol was prescribed for post operative pain relief.

## Discussion

Anaesthetic and postoperative care for patients with learning disabilities can be very challenging for all members of the operative team. There have been many reports describing perioperative care of patients with various genetic syndromes but the majority of them have been related to paediatric patients. Adult patients with genetic syndromes and learning disabilities, apart from specific features associated with the syndrome, present additional challenges for the anaesthetic team because of associated medical disease and potential challenging behaviour against medical staff.

The genetics of DOOR syndrome has been described in detail elsewhere [2,7]. In the majority of cases reported, an autosomal recessive inheritance is proposed. This hypothesis is supported mainly by the work of Rajab et al. [7], who described a family with four children affected by this syndrome. However, some cases have appeared to be inherited in different patterns: autosomal dominant, pseudodominant or X-linked. Autosomal dominant inheritance is usually associated with mild to moderate learning disability only. DOOR syndrome has been also associated with some biochemical abnormities. Increased levels of 2-oxoglutarate in the urine have been described due to decreased activity of 2-oxoglutaratedecarboxylase (E1_0_) in both fibroblasts and white blood cells [6].

Central nervous system and musculoskeletal system pathologies are a prominent feature of DOOR syndrome. They have very important implications for the anaesthesiologist involved in the perioperative management of these patients.

Typical facial features are an important part in the diagnosis of DOOR syndrome. They include a coarse face with shortened bifrontal diameter, broad nasal bridge, long prominent philtrum, high arched palate with thick palatine ridges and low set ears. These features may render nasotracheal intubation impossible and orotracheal intubation difficult. Poor patient co-operation, deafness and intellectual disability make awake fibre optic intubation unfeasible. Plans for anticipated difficult airway management, including tracheal intubation through a supraglottic device or use of video-laryngoscope, should be implemented [8]. In patients with no aspiration risk and for short procedures, a supraglottic airway alone may be an option for airway management. Appropriate premedication should be used. We have good experience in the use of buccally administered midazolam (dose 0.15-0.25 mg.kg^−1^) [9]. Epilepsy is a common feature of this syndrome. Affected individuals usually develop serious seizures early in their life. The seizures are usually of a progressive nature, with increasing frequency or severity. Episodes of status epilepticus are common and the seizures can occur on a daily basis [4]. With antiepileptic medication, liver cytochrome P450 systems may be induced (CYP3A4 is induced by both carbamazepine and phenytoin). Doses of opioids and benzodiazepines should, therefore, be properly adjusted^10^. Seizure control and antiepileptic medication should be optimized before anaesthesia. In our patient, we used sevoflurane as a volatile agent at concentration of 1 MAC. This approach may be controversial because other volatile agents like isoflurane and desflurane have been described to have an anti-seizure potential. On the other hand, we have good experience with sevoflurane and this agent is not contraindicated in epilepsy. In low concentrations and when supplemented with benzodiazepines, nitrous oxide and opioids it has no significant pro-epileptic activity [11]. Other central nervous system abnormities include deafness, which may be either sensoneurinal congenital or progressive and optic atrophy leading to blindness [4]. Varying degrees of cerebral palsy may also be present.

Congenital cardiac defects have been found in approximately 20% of reported cases of DOOR syndrome. Abnormities include patent ductus arteriosus, atrial and ventricular septal defects [4,5]. Careful cardiac evaluation, auscultation, ECG and echocardiography in indicated patients should be undertaken before general anaesthesia.

Adult patients with DOOR syndrome are very often obese. The main contributory factor is patient immobility. Obesity is associated with well-known pathophysiological consequences; delayed gastric emptying, high metabolic demand, decreased functional residual capacity of lungs, cardiovascular pathologies (increased cardiac output, increased blood volume, hypertrophy of left ventricle, pulmonary hypertension) and a high incidence of obstructive sleep apnoea. It is possible that in our patient some of his associated symptoms, mainly gastroesophageal reflux, asthma and hypertension could have been related to undiagnosed sleep apnoea. If using the STOP-BANG questionnaire [12], the patient would have at least 3 or 4 risk points for sleep apnoea.

Other systemic anomalies have been documented in association with DOOR syndrome. Dental findings range from hypoplastic enamel to wide spacing, abnormal size, shape or number of teeth [2]. Genito-urinary abnormities are variable and may include one-sided renal agenesis, duplicated kidney, cystic and dysplastic kidneys, hydroureteronephrosis without obstruction or frequent urinary tract infections leading to urosepsis [4,5]. Renal function should be assessed before general anaesthesia.

## Conclusion

DOOR syndrome poses many challenges to the anaesthesiologist: it is a multisystem disorder in patients with moderate to severe intellectual disability and there is a distinct possibility of a difficult airway. Careful planning and perioperative management is essential to overcome these obstacles.

## List of abbreviations

BMI: body mass index
cm: centimetres
DOOR syndrome: Congenital **D**eafness, **O**nychodystrophy, **O**steodystrophy and Mental **R**etardation
ECG: electrocardiogram
Kg: kilograms
mcg: micrograms
mg: milligrams
RAE tube: curved tube used for ENT and oral surgery procedures named after the inventors Ring, Adair and Elwyn
RE: response entropy
SE: state entropy
